# Revealing Sources of Variation for Reproducible Imaging of Protein Assemblies by Electron Microscopy

**DOI:** 10.3390/mi11030251

**Published:** 2020-02-27

**Authors:** Ibolya E. Kepiro, Brunello Nardone, Anton Page, Maxim G Ryadnov

**Affiliations:** 1 National Physical Laboratory, Teddington, Middlesex TW11 0LW, UK; 2 CEM Corporation, Buckingham Industrial Park, Buckingham MK18 1WA, UK; 3 Biomedical Imaging Unit, Faculty of Medicine, University of Southampton, Southampton SO16 6YD, UK

**Keywords:** protein self-assembly, protein filaments, virus-like capsids, electron microscopy, negative staining

## Abstract

Electron microscopy plays an important role in the analysis of functional nano-to-microstructures. Substrates and staining procedures present common sources of variation for the analysis. However, systematic investigations on the impact of these sources on data interpretation are lacking. Here we pinpoint key determinants associated with reproducibility issues in the imaging of archetypal protein assemblies, protein shells, and filaments. The effect of staining on the morphological characteristics of the assemblies was assessed to reveal differential features for anisotropic (filaments) and isotropic (shells) forms. Commercial substrates and coatings under the same staining conditions gave comparable results for the same model assembly, while highlighting intrinsic sample variations including the density and heterogenous distribution of assemblies on the substrate surface. With no aberrant or disrupted structures observed, and putative artefacts limited to substrate-associated markings, the study emphasizes that reproducible imaging must correlate with an optimal combination of substrate stability, stain homogeneity, accelerating voltage, and magnification.

## 1. Introduction

Biological processes and systems employ micromachines to deliver a variety of functions ranging from intra- and inter-cellular gene transfer [1] to antimicrobial host defense [2] and tissue repair [3]. These are hierarchical supramolecular structures that assemble from perfectly matched subunits or building blocks. The reproducibility of their assembly continues to inspire artificial designs, which aim to acquire a level of control over a desired function and often explore properties that are not characteristic of their naturally occurring counterparts [4]. However, be it a virus or an extracellular scaffold, synthetic micromachines use chemically identical protein materials and rely on the correct folding of protein subunits along with their ability to effectively complex with other biomolecules, e.g., nucleic acids. Therefore, the characterization of protein assemblies comes down to the ability to detect the same chemistry, which provides a basis for developing routine procedures for sample preparation and measurement. Nevertheless, variations in analysis persist [5].

This is particularly the case for measurements by electron microscopy that require the deposition of the assembled material on a substrate, and the use of contrast agents (stains) to highlight morphological and structural features of the specimen in question [6]. An increasingly important approach is the use of correlated datasets and cross-comparison of data obtained by different imaging modalities (e.g., electron and super-resolution microscopy), with chemical imaging (Raman, mass-spectrometry) providing an additional level of detail and complexity. Taken together, the current requirements rely on optimized and reproducible protocols for sample analysis as well as capability combinations that can support correlated imaging [7]. Improving reproducibility of imaging will also benefit existing practices in the National healthcare systems, e.g., in the differential diagnosis of neurodegenerative diseases. In long run, these will help meet regulatory criteria for the traceability of measurement results, which accompany the development of advanced therapies (e.g., gene therapy) and imaging modalities.

Yet, despite all this progress and the appreciation of data variability, systematic attempts to address the impact major sources of variation have on data interpretation are lacking. Herein we report a study that fills this gap by investigating the impact of substrate and stain variations on the analysis of the most common morphologies of protein micromachinery—capsid-like shells and filamentous structures.

## 2. Materials and Methods

### 2.1. Protein Assembly Preparations

Models 1, 3, and 4 were assembled from synthetic polypeptide constructs prepared as reported elsewhere [8,9,10]. Briefly, monomer units were synthesized on a Liberty Blue microwave peptide synthesizer (CEM Corp., Matthews, NC, USA) on a Rink amide 4-methylbenzhydrylamine resin (model 1) and a Fmoc-Gln(Trt)-Wang resin (models 3 and 4) using 2-(1H-benzotriazol-1-yl)-1,1,3,3-tetramethyluronium hexafluorophosphate (HBTU) and diisopropylethylamine as coupling reagents and conventional solid-phase Fmoc/*t*Bu protocols. Fmoc-Lys(Mtt)-OH was used to enable orthogonal conjugation for model 1 [2] and Fmoc-Lys(Alloc)-OH was used to label model 4 with Alexa Fluor 488 [8]. After cleavage from the resin and deprotection, the synthesized constructs were purified by reversed-phase high performance liquid chromatography (RP-HPLC) and were then lyophilized and had their identities confirmed by matrix-assisted laser desorption/ionization time of flight (MALDI-ToF) mass spectrometry. The purified materials were used to prepare aqueous solutions (200 μL, 10–300 μM) in filtered (0.22 μm) 10 mM 4-morpholinepropanesulfonic acid (MOPS), as well as pH 7.4, and were left for incubation over at least five hours. Model 4 samples for fluorescence microscopy were prepared by incubating model 4 with its Alexa 488 labelled derivative at a molar ratio of 1:10^−3^. Model 2 was purchased from ATCC (*Escherichia coli* bacteriophage MS2, ATCC 15597-B1^TM^) and cultured according to the proprietary protocols. After incubation all materials were used for imaging.

### 2.2. Electron Microscopy

The assembled materials with 8 μL drops of the solutions were applied to specimen grids as described in the text. After 1 min, the excess solvent was removed using blotting paper and the grids were stained either with uranyl acetate (aq. 2%, w/v) or Nano-W (Nanoprobes, aq. 2% w/v) for 10–30 sec before blotting the excess stain. Electron micrographs were recorded using transmission electron microscopy (TEM) instruments: JEOL 1010 equipped with Orius SC1000 charged coupled device camera (Gatan Inc., Pleasanton, CA, USA), JEM1400-Plus equipped with an OneView 4K camera (Gatan Inc., Pleasanton, CA, USA), and Tecnai (FEI Company, Hillsboro, OR, USA) T12 equipped with a Morada G2 camera (EMSIS GmbH, Münster, Germany). Typical dose conditions were a few e^−^/Å^2^ at exposure time of 500 ms–4 sec, with the accelerating voltage of 80–120 kV.

### 2.3. Specimen Grids and Coatings for Transmission Electron Microscopy

All specimen grids, substrates and coatings, were purchased and used following glow discharge, except graphene coatings [11]: carbon coated copper grids, copper grids coated with Formvar and carbon films, copper grids coated with Formvar and silicon monoxide coatings and silicon nitride TEM window (all from Electron Microscopy Sciences), Quantifoil carbon gold coated with a single graphene layer (Agar Scientific Ltd.), and copper grids coated with holey carbon with graphene oxide coatings (EM Resolutions Ltd.). In selecting substrates, consideration was given to the thickness of coating films, surface background contrast, conductivity, and hydrophilicity. For example, Formvar coated grids stabilized with evaporated carbon (1–30 nm) were chosen due to their high mechanical stability. Silicon monoxide provides both low background contrast and high stability under the electron beam and is more hydrophilic than the carbon film. Holey carbon coated films comprising of different sizes holes (up to 100 μm) provide increased image contrast in the hole regions, which is unlike Quantifoil^®^: perforated support foils that have pre-arranged holes of regular sizes and shapes. Graphene films are deposited as a single carbon atomic layer and provide mechanically strong, chemically inert, and electrically and thermally conductive coatings. These films are available in single-layer (0.34 nm) and multi-layer formats (0.7–2.8 nm). Graphene oxide (GO) films (~1 nm in thickness) used as hydrophilic support coatings on copper or gold grids are nearly transparent to the electron beam offering better background contrast compared to graphene. Key characteristics of the substrates and coatings used in the study are given in Table 1.

### 2.4. Fluorescence Microscopy Imaging

Samples were incubated over five hours and imaged in 8-well chambers (IBIDI μ-Slide with #1.5H D263M Schott glass bottom) at 200 μL per well using an Olympus IX81 inverted confocal microscope with 60x oil immersion objective (UPSAPO 60X, NA 1.35, WD 0.15 mm).

## 3. Results and Discussion

This study employs two main types of self-assembled protein structures. One type, spherical capsid-like shells, is exemplified by model 1, a virus-like capsid assembled from a re-engineered antimicrobial domain of human breast milk protein lactoferrin [8], and model 2, the capsids of the MS2 bacteriophage. The other type is represented by protein filaments of different lengths, which are exemplified in models 3 and 4 [9,10,12]. The models of the first type share archetypal physicochemical characteristics by being hollow nanoparticles of uniform morphology. Both systems allow a degree of polymorphism and can vary by size, but not form. The assembly of models 3 and 4 is based on the same protein folding motif, an α-helical coiled coil [13]. The models are longitudinal propagations of coiled-coil subunits that bundle up with the pre-defined degrees of lateral association resulting in two uniform filament diameters.

### 3.1. Effects of Contrast Staining: Staining Patterns

**Figure 1:**
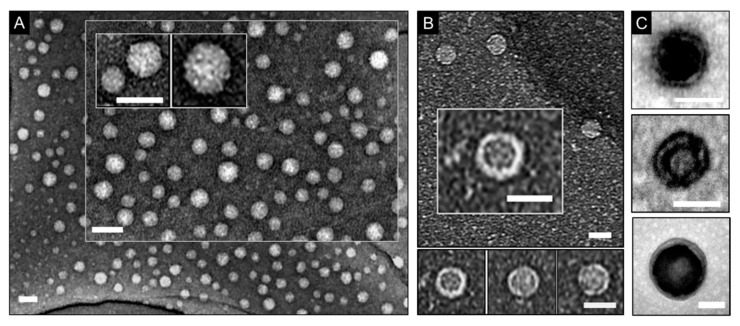
Contrast staining in imaging spherical, shell-like protein morphologies. Electron micrographs of model 1 (**A**), model 2 (**B**), and (**C**) model 1 on copper grids coated with holey carbon, and graphene oxide films (**A**,**B**) and carbon film (**C**). Scale bars are 50 nm (**A**,**C**) and 30 nm (**B**). Staining conditions: Nano-W^TM^ (aq. 2%, w/v) for (**A**,**B**) and (**C**, top and bottom), and uranyl acetate (aq. 2%, w/v) for (**C**, middle).

As gauged by TEM, models 1 and 2 were morphologically discrete spheroids with a characteristic negative staining pattern (Figure 1A). The individual spheroids appeared having darker centers indicating hollow interiors (Figure 1B,C). This is the hallmark of protein shells, cages, and capsids that is expected for the stains used [14,15]. Consistent with observations by others [14,15,16,17], variations in staining were due to the accumulation of stain in the interior of the spheroids, which contrasts with solid nanoparticles assembled through condensation with nucleic acids [18].

Dehydration did not prove to be a contributing factor for the variations; samples imaged immediately after staining showed the same patterns as samples left to air dry over several hours and days. In part, this can be explained by the stability of the morphologies and the density of their shells limiting the impregnation of the stains’ heavy metals. As a result, only collapsed or open spheroids showed evidence of hollow interiors (Figure 1B,C).

Model 1 comprises of two layers of β-pleated networks arranging into a double-wall shell [8]. This may explain the alternation of stain patterns observed in individual spheroids, suggesting a double wall structure (Figure 1C). Multi-walled structures, which could provide denser assemblies, cannot be excluded for this model entirely either. The protein subunits of model 2 form a shell with contiguous, but thinner, walls (21–25 Å in thickness), which also renders them more permissive to stains [15]. Thus, selective staining of hollow shells or shell-like structures by applying a negative stain remains challenging as both staining scenarios can occur simultaneously in the same sample. Negative staining applied to the surrounding environment of the analyzed specimen eases the crossing of the material by the electron beam, which results in a darker background around the specimen. This is unlike variations in staining that appear to resemble positive staining patterns and by seemingly enhancing the contrast of the analyzed material itself. The interiors of hollow shells or spaces between walls in bi- or multi-layered shells serve as a background for the protein material that makes up the shell. If the stain visibly accumulates in this background its contrast becomes darker i.e., enhanced. Similar effects are observed during the intracellular imaging of sectioned cells transfected with shells (e.g., viruses,) that can traverse cell membranes [19]. However, given that the background for the shells is no longer a grid substrate, but a cell, contrast of shell interiors may appear shaper and more readily distinguishable.

**Figure 2:**
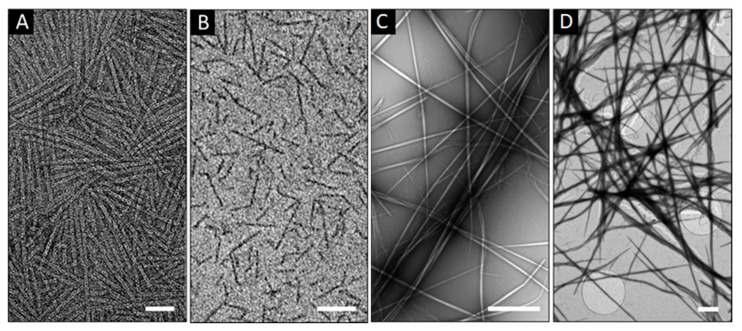
Staining patterns for filamentous protein assemblies. Electron micrograph of negatively stained model 3 (**A**) on silicon nitride TEM window, with staining variations that appear positive (**B**) on carbon film on copper grids (**B**). Electron micrographs of model 4, negatively stained on copper grids coated with Formvar and carbon films (**C**) and positively stained on Quantifoil^®^ gold grids coated with a single graphene layer (**D**). Scale bars are 50 nm (**A**,**B**) and 1 μm (**C**,**D**). Staining conditions: uranyl acetate (aq. 2%, w/v).

Variations in staining can also be used as differentiating factors for the imaging of filamentous protein assemblies. Tubular structures can be characterized by the appearance of similar dark–light patterns, which is not as common for fibrillar structures that usually give homogenous patterns for negative staining (Figure 2A,B). Since fibers are often bundles of thinner filaments, thickening can bring up contributions of negative and positive contrasts within a single fiber, revealing individual constituent filaments [20]. This may help distinguish between well packed and amorphous fibrillar structures, with the latter incorporating patterns that may appear positive. Extended, micron-long fibers need imaging at low magnifications, at which a non-homogenous stain coverage can become more apparent (Figure 2C). This is due to more stain accumulating in the sites of higher fiber density, e.g., due to fiber crossing, entangling or stacking (Figure 2C,D). Such higher local densities may appear as positively stained. Therefore, this effect predominantly impacts on negatively stained samples, in which the expectedly darker contrast of the environment, which surrounds individual fibers, allows for visualizing them clearly, whereas the enhancement of the contrast in the sites of crossed or entangled fibers can make the staining of individual fibers indistinguishable from that of their background (Figure 2D).

The reverse scenario of positive staining is less subject to these variations as stain accumulates in the fibers themselves and merely gives enhanced contrast for sites of higher protein density. Thus, simultaneous positive and negative staining that is often characteristic of hollow spheroid structures is common for long, wispy, and bundled filamentous structures, but have a greater impact on the interpretation of imaging results at low resolution. In this regard, perhaps the most important factor for the quality of imaging is to maintain the homogenous stain coverage of the specimen. Among the stains available for electron microscopy, uranyl acetate (UA) represents a popular choice for sample optimization as it effectively hydrates the specimen and rapidly crystalizes with the formation of a single crystalline film-like layer. In solution, the stain comprises of co-existing ionic complexes without a charge preference, that are able to bind to anionic and cationic moieties [21]. Other stains (e.g., phosphotungstic acid, Nano-W^TM^, or ammonium molybdate) can also offer improvements in contrast enhancement. However, the use of all stains is based on the same principles of heavy metal crystallization, which limits improvements to the ability of each stain to form a homogeneous film on the substrate. This relates to how efficient negative staining is in preserving the native structure of the specimen, which limits imaging resolution to a maximum of 1.8–2 nm [22,23]. Regardless, UA proves to give an optimum combination of high and differential image contrasts at low aqueous concentrations (1–2%), with its thin films having mass thickness significantly higher than that of other stains [24]. Therefore, other compounding factors impact on the quality of differential staining which is dependent on the quality of stain solutions, pH, and electrical charge of both the stain and the sample as heavy ions typically crystalize by reacting with nucleophilic groups (e.g., anionic amino acids, amino groups, phosphate groups). Therefore, residual buffer salts, especially phosphates, in the sample can catalyze crystallization leading to excessive mass thicknesses masking the specimen. This dependence may also explain the cases of overstaining for overlapping or clustering fibers [9,24], which cannot be excluded completely for matrix- or network-like fibrillar assemblies [25]. Conversely, homogenous staining of individual fibers may help differentiate branched or split fibers from intertwined or overlapping structures [26].

### 3.2. Effects of Substrate: Nature and Homogeneity of Substrate Surface

**Figure 3:**
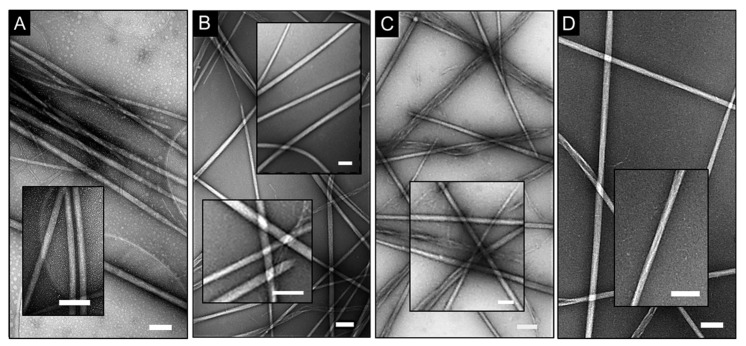
Negative staining on different substrates. Electron micrograph of model 4 on copper grids coated with holey carbon and graphene oxide (**A**), Formvar and carbon films (**B**), Formvar and silicon monoxide coatings (**C**) and silicon nitride TEM window (**D**). Scale bars are 250 nm. Insets highlight cross-sectioned fibers. Staining conditions: uranyl acetate (aq. 2%, w/v).

Despite the important role that electron microscopy plays in life sciences, there is scarce information with regards to empirical variations in the analysis of protein assemblies depending on substrates. The impact of different substrate coatings, which are homogenous thin films, on electron microscopy grids is often acknowledged and cannot be underestimated. Given that sources of irreproducibility may originate from differences in the chemical nature of the substrates (e.g., thickness, hydrophobicity, and surface charges), we tested different formulations under the same staining conditions for the same self-assembly model. Contrast patterns in obtained electron micrographs for model 4 proved to be comparable across the coatings used (Table 1), while negative staining was apparent for individual fibers (Figure 3).

Some coatings, such as graphene oxide, can carry a negative charge. Negatively stained particulate patterns were observed on the coatings, which suggests debris particles (Figure 3A). Although these can be attributed to small pre-fibrillar oligomers, which are in equilibrium with protein assemblies in solution [10], mechanical artefacts due to the electron beam treatment of the coating surface cannot be excluded. Model 4 is indeed strongly cationic, and the adsorption of the particles on the negative surfaces can be expected (Figure 3A). An alternative scenario of coating rupture caused by electron beam would be possible if the particulate patterns were apparent only in the background of the specimen. Instead, these were visible across the whole surface including fibers, suggesting negatively stained protein material, as was also observed for shell-like assemblies (Figure 1B). In addition, the cross-sections of overlapping fibers in all images appeared as positively stained, which is consistent with the effects discussed earlier.

**Figure 4:**
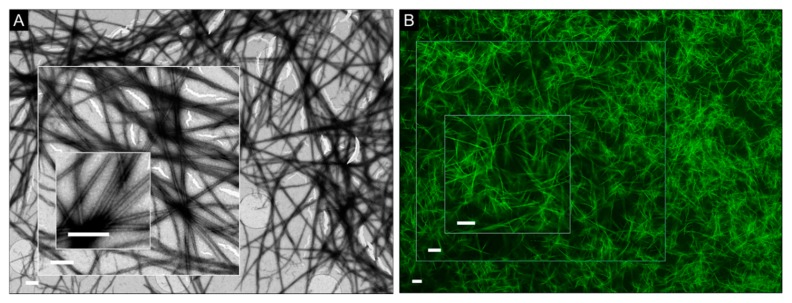
Effect of coating damage during imaging. (**A**) Electron micrograph of model 4 on Quantifoil^®^ grids coated with a single graphene layer (0.34 nm). (**B**) Fluorescent micrographs for model 4 assembled at the same conditions are given for visual comparison. Scale bars are 0.8 μm (**A**) and 5 μm (**B**). Staining conditions: (**A**) uranyl acetate (aq. 2%, w/v).

Interestingly however, in some cases of crossed fiber pairs, contrast was enhanced for one of the fibers (Figure 3B–D). It is likely to be an artefact since the stain accumulates at the edges of the fiber that runs on top of the other, thus creating a localized contrast background, which appears enhanced. The effect did not seem to depend on the nature of the substrate, but was more evident for coatings and alternative non-carbon substrates which are chemically inert, mechanically stable, and are able to withstand multi-step sample processing (Figure 3B,D). More resilient substrates (e.g., silicon nitride) can operate at a wider range of temperatures when compared to carbon-coated grids, which makes them particularly versatile for experiments performed at high accelerating voltages. Common flaws during imaging on carbon films, which can contribute to artefacts, owe to their tendency to tear and crack. This can be due to mechanical ruptures and as a result of direct damage by electron beam radiation [27,28]. This problem remains largely unsolved and is especially important for applications that use atomically thin layers to reach the most homogenous film coverage available. To emulate such a requirement, we imaged model 4 fibers on carbon-based grids coated with a single graphene layer (0.34 nm). Although micrographs of quality and detail comparable to those of other substrates were obtained, the coating could not withstand mild experimental conditions, e.g., accelerating voltage of 80 kV (Figure 4). Thus, the choice of a coating is a matter of compromise between the substrate stability, background homogeneity, and the precision of measurement.

The same imaging constrains hold true for spheroid or shell-like protein assemblies. Substrate variations have a lesser impact on these structures individually, as they tend to be much smaller when compared to extended fibers. The impact of the substrate on protein assemblies is more evident at low magnifications. For spherical assemblies, these provide access to more statistically significant data, which is critical for obtaining other important properties including density, polydispersity, and size distribution. Yet, contrast variations remain the most important problem that continues to beset even the seemingly straightforward imaging of protein capsids [29]. Cryo-EM can complement negative staining microscopy and indeed in combination these two methods help resolve hollow capsids-like structures [30]. With cryo-EM reconstructions providing an attainable resolution in the 5–10 nm range [31], the problem is largely unsolved prompting the development of more effective staining procedures, while the pursuit for an optimal substrate for cryo-EM remains unfulfilled [32].

## 4. Conclusions

In summary, this case study has probed the impact of substrates and staining—two arguably most common sources of variation—on the imaging of protein assemblies by electron microscopy. Two basic supramolecular morphologies, exemplified by previously reported assemblies, were investigated. Namely, hollow shells and filamentous structures. Two exemplar models for each of the morphologies were chosen based on their size differences, diameter and persistence length, and the sampling density. Such differentiators were necessary for the cross analysis of the models at low and high magnification scales. Variabilities of environmental conditions, in which the structures were assembled, were minimized by using identical assembly conditions, which was also consistent with that the models share similar physicochemical properties (e.g., pI, hydrophobicity, stability, folding). This experimental design allowed us to reveal the morphology of resulting assemblies depending on the two sources of variation. None of the imaged samples showed signs of aberrant, distorted, or disrupted assemblies. Even in cases of substrate damage, the assemblies remained intact and apparent for analysis. Variations in contrast could be ascertained, but these mainly concerned differential staining patterns as a result of varied background due to fiber entangling and stain accumulation in the interior of the capsid-like shells. Different substrate coatings were found to perform according to their manufactured characteristics. Surface charge did not appear to have a significant impact on image quality, except for graphene oxide coatings that were associated with particulate patterns which suggests an artefact contribution. The results support an overall conclusion that the stability of the chosen substrate and coating, homogeneity of background staining, and the level of precision for the required measurements are the main determinants for reproducible imaging. Patterns of seemingly positive staining in negatively stained samples strongly depend on the type and morphology of a given assembly and its physicochemical characteristics. Improvements in sample preparation include a better control over the sampling density, which is likely to work best for relatively small and monodisperse structures [33], and an aligned orientation of anisotropic assemblies (e.g., fibers or filamentous viruses under a shear flow), which might however cause amyloid-like aggregation effects leading to artefacts [34]. All in all, when choosing an imaging strategy, one should critically consider empirical correlations between the properties of the biological system in question with imaging conditions. Since there is a growing need for imaging biological processes at the nanoscale and in 3D, which is increasingly attempted to be addressed by in situ TEM [35], progress towards more effective experimental approaches that can minimize the outlined and other sources of irreproducibility are more important than ever.

## Figures and Tables

**Table 1:** Substrate and supporting coatings used in the study.

Substrate	Surface Charge	Thickness (nm)
Carbon film ^a^	negative ^b^	~5–6
Formvar/carbon films ^a^	negative ^b^	~10/5–6
Formvar/silicon monoxide coatings ^a^	negative ^b^	~10/5–6
Silicon nitride TEM window	positive ^b^	10
Single graphene layer ^c^	negative	~0.34
Holey carbon/graphene oxide coatings ^a^	negative	~5–6/1

^a^ on copper grid; ^b^ glow discharged [11]; ^c^ on Quantifoil gold grids.
